# A comparison of Bayesian and frequentist approaches to incorporating clinical and biological information for the prediction of response to standardized pediatric colitis therapy

**DOI:** 10.1371/journal.pone.0295814

**Published:** 2024-03-06

**Authors:** Zhu Wang, Jia Nie, Xing Song, Lee A. Denson, Jeffrey S. Hyams

**Affiliations:** 1 Department of Preventive Medicine, University of Tennessee Health Science Center, Memphis, TN, United States of America; 2 Barshop Institute for Longevity and Aging Studies, University of Texas Health Science Center at San Antonio, San Antonio, TX, United States of America; 3 Health Management and Informatics, University of Missouri, Columbia, MO, United States of America; 4 Cincinnati Children’s Hospital and Medical Center, Cincinnati, OH, United States of America; 5 Connecticut Children’s Medical Center, Hartford, CT, United States of America; Sapporo Medical University, JAPAN

## Abstract

**Background:**

The prospective cohort study PROTECT is the largest study in pediatric ulcerative colitis (UC) with standardized treatments, providing valuable data for predicting clinical outcomes. PROTECT and previous studies have identified characteristics associated with clinical outcomes. In this study, we aimed to compare predictive modeling between Bayesian analysis including machine learning and frequentist analysis.

**Methods:**

The key outcomes for this analysis were week 4, 12 and 52 corticosteroid (CS)-free remission following standardized treatment from diagnosis. We developed predictive modeling with multivariable Bayesian logistic regression (BLR), Bayesian additive regression trees (BART) and frequentist logistic regression (FLR). The effect estimate of each risk factor was estimated and compared between the BLR and FLR models. The predictive performance of the models was assessed including area under curve (AUC) of the receiver operating characteristic (ROC) curve. Ten-fold cross-validation was performed for internal validation of the models. The estimation contained 95% credible (or confidence) interval (CI).

**Results:**

The statistically significant associations between the risk factors and early or late outcomes were consistent between all BLR and FLR models. The model performance was similar while BLR and BART models had narrower credible intervals of AUCs. To predict week 4 CS-free remission, the BLR model had AUC of 0.69 (95% CI 0.67–0.70), the BART model had AUC of 0.70 (0.67–0.72), and the FLR had AUC of 0.70 (0.65–0.76). To predict week 12 CS-free remission, the BLR model had AUC of 0.78 (0.77–0.79), the BART model had AUC of 0.78 (0.77–0.79), and the FLR model had AUC of 0.79 (0.74–0.83). To predict week 52 CS-free remission, the BLR model had AUC of 0.69 (0.68–0.70), the BART model had AUC of 0.69 (0.67–0.70), and the FLR model had AUC of 0.69 (0.64–0.74). The BART model identified nonlinear associations.

**Conclusions:**

BLR and BART models had intuitive interpretation on interval estimation, better precision in estimating the AUC and can be alternatives for predicting clinical outcomes in pediatric patients with UC. BART model can estimate nonlinear nonparametric association.

## Introduction

Outcomes following current standard therapies for children with ulcerative colitis (UC) are difficult to predict because of extensive and severe disease at diagnosis in the majority of affected individuals. It has been noted that age at diagnosis, anatomical extent and disease course all contribute to a wide range of outcomes with some children quickly responding to therapy and remaining in clinical remission, some who are refractory to all medical interventions and who go to colectomy, and the majority who have a waxing and waning course [1–5]. Whereas most adults with UC tend to have more limited disease and often respond nicely to oral mesalamine, less than half of all children have a similar course with most requiring corticosteroids or advanced therapies such as biologics [6–9].

The NIH supported PROTECT study (Predicting Response to Standardized Pediatric Colitis Therapy (U01DK095745)), initiated in 2012 and completed in 2016, is the largest study in pediatric UC with standardized treatments, and has provided valuable data to help develop predictive models of clinical outcomes of pediatric UC. By concomitantly obtaining biospecimens for translational studies at diagnosis and during the first year following therapy along with highly standardized clinical data, it facilitated a better understanding of inter-patient variability in response to therapy and provided insights into the pathways that sustain colonic inflammation [9]. In this large, multicenter inception cohort, the primary goal was to identify predictors of corticosteroid (CS)-free remission achieved with mesalamine maintenance therapy following initial treatment with mesalamine or CS.

In this article, we compared Bayesian and frequentist approach to predict clinical outcomes using data from the PROTECT study. FLR is a typical choice in many applications [8, 9]. In the frequentist approach, the parameter of interest (e.g., an odds ratio) is considered a fixed number. The Bayesian approach, however, treats the parameters of interest as random variables, and, therefore, parameters can be described with probability distributions [10]. The frequentist confidence interval (CI) has a long-run frequency interpretation. As an example, an interpretation follows: we can be 95% confident that the true (unknown) estimate of odds ratio of baseline Mayo < 10 for week 4 remission would lie within the CI (1.11, 3.12), based on hypothesized repeated experiment. On the other hand, the Bayesian confidence interval (or more formally credible interval, CI) can be interpreted in a probabilistic way. For instance, one can interpret a Bayesian CI as follows: with 95% probability, the odds ratio of baseline clinical severity Mayo score < 10 for week 4 remission is between 1.11 and 3.12. This would be a more natural interpretation for clinicians [11]. We utilized two Bayesian models. In a BLR, we incorporated the external information via informative priors. As a new machine learning approach, BART can take informative priors when constructing regression trees [12]. We intended to fill the knowledge gap of a lack of comparative study to evaluating BLR, BART and FLR predictive models for pediatric patients with UC.

## Methods

### Study population

In the PROTECT Study, patients were recruited from 29 North American centers between July 2012 and April 2015. Children from age 4 to 17 years inclusive with a clinical history consistent with colonic inflammation (any combination of diarrhea, bleeding, abdominal pain) were eligible for study. Inclusion criteria, clinical assessments for the determination of clinical variables and demographic information for patients enrolled in this study have been described before [8, 9]. Participants were enrolled and completed all baseline assessments prior to initiation of therapy and were followed for a minimum of 1 year, through April 2016. The data includes post-baseline assessments at 4 and 12 weeks, along with interim phone calls, visits, and hospitalizations as needed. Visit assessments included Pediatric UC Activity Index (PUCAI), partial Mayo activity score (excluding the endoscopy sub-score), clinical evaluation, and standard-of-care clinical labs. Stool samples and plasma for specialized laboratory assessments were collected at 4 and 12 weeks. Depending upon initial PUCAI score, patients were initially treated with either mesalamine (mild disease), oral CS (moderate disease), or intravenous (IV) CS (severe disease) based on standardized guidelines but with some physician discretion allowed. We followed the same per-protocol population approach as in the original study [8, 9]. The authors had no access to information that could identify individual participants during or after data collection. The data were accessed on March 10, 2022. This research has been approved by the Institutional Review Board of University of Texas Health Science at San Antonio and University of Tennessee Health Science Center. All methods were carried out in accordance with relevant guidelines and regulations. Informed consent or assent from a parent and/or legal guardian for study participation was obtained.

### Outcomes

The primary outcome was week 52 CS-free remission defined as clinical remission (PUCAI score of <10) with no corticosteroid (CS) use for 4 weeks or longer immediately before week 52, no medical therapy beyond mesalamine, and no colectomy. The secondary late outcome was escalation to anti-TNFα therapy at any time in the 52 weeks. Early outcomes were week 4 and week 12 CS-free remission. Week 12 CS-free remission was similar to the primary late outcome except for no CS for a minimum of 2 weeks. Week 4 CS-free remission was defined over 4 weeks.

### Predictors

We focused on pre-determined predictors of clinical outcomes [8, 9]. These predictors were utilized with different predictive models. When missing data were present, we focused on the complete data analysis for the early outcomes as in [8]. For the late outcomes, imputation-based approaches were presented as in [9] with additional technical details for the associated statistical models below.

### Predictive models

We developed predictive models with three different statistical methods described below for each outcome. Adjusted odds ratios were presented when applicable. We assessed model fit with the AUC of the ROC curve and corresponding 95% credible or confidence interval (CI), sensitivity, specificity, positive predictive value, negative predictive value and corresponding 95% CI. The cut-off value was typically chosen at a predicted probability of 0.50. However, for the use of additional medical therapy or colectomy by week 4 on the intravenous corticosteriod group, a smaller number 0.39 was chosen to obtain meaningful results. We also assessed 10-fold cross-validated AUC (CV-AUC) and 95% CI, which was different from the bootstrapping in [8] or leave-one-out cross-validation in [9]. The last two approaches are computationally intractable in the Bayesian framework, which is the focus of this article. Ten-fold cross validation randomly split the data into 10 disjointly equal subsets and repeatedly used nine subsets for model fitting and the remaining subset for validating AUC. The calculation of CV-AUC is further detailed when the specific methods are described below. For the early outcomes, we conducted separate modeling within initial treatment group (mesalamine, oral CS, IV CS). For the late outcomes, separate modeling was developed for patients with biological data. Analyses performed using R version 4.1.3 (2022-03-10), package rstanarm version 2.21.3 and BART version 2.9.

### Methods 1 –Bayesian logistic regression

Unlike the frequentist logistic regression, Bayesian method assumed that the coefficients followed some prior distributions. It was also assumed independence among the priors. Throughout, we utilized the weakly informative priors [10]. Selecting prior distributions is a critical step in Bayesian analysis, and we chose weakly informative priors by utilizing the default prior configurations provided in the R package rstanarm, as recommended in [10]. These priors are specifically designed to capture general prior uncertainty across a range of reasonable parameter values and are tuned with information from the data. Unless we possess strong prior information, opting for the defaults in rstanarm generally results in more stable simulation outcomes compared to attempting to tune other arbitrary priors. For each logistic regression model, we ran Markov chain Monte Carlo (MCMC) with 4 chains where each chain contained 10,000 iterations with the first half for burn-in and assessed convergence visually. We also computed quantitative convergence diagnostics, such as the Gelman-Rubin statistic and effective sample size (ESS) to assess the convergence of MCMC simulations. The choice of the specific number of burn-in iterations, such as 5,000 in our analysis, is often determined empirically based on the behavior of the chain and may vary depending on the complexity of the model and the specific MCMC algorithm used. The results were presented as odds ratios using the 20,000 posterior draws for each logistic regression coefficient and their 95% CIs. Model performance was evaluated similarly using the posterior draws. In the 10-fold cross-validation procedure, we repeatedly built Bayesian logistic regression with nine subsets of data and obtained distribution of AUC from the remaining testing data. The AUC scores from all folds were pooled to derive a posterior distribution of the AUC, which was then used to calculate the mean and 95% credible intervals of the AUC.

For the late outcomes, we utilized 100 multiple imputations and combined the results following the recommended strategy in [13]: (i) simulate many draws from the posterior distribution in each imputed dataset, and (ii) mix all the draws. The mixed draws approximate the posterior distribution. For each logistic regression model, this approach led to 100×20,000 posterior draws, from which, odds ratios were computed, as well as the 95% CIs. Model performance was evaluated similarly. In the CV-AUC procedure, we obtained AUC distribution for each fold following [13] again and took average of cross-validated AUC and confidence intervals. Furthermore, to evaluate the posterior predictive accuracy of a Bayesian regression model, we computed expected log-predictive density (ELPD). The higher the ELPD, the better a model since higher ELPDs indicate greater posterior predictive accuracy to predict new data points. The ELPD was estimated by leave-one-out cross validation. With multiple-imputations, we implemented the following procedure: we obtained pointwise ELPD for each multiple-imputed dataset, computed means of pointwise predictive densities, where means were over all imputed datasets. This resulted in pointwise predictive densities. A model’s ELPD was then sum of log of pointwise predictions.

### Method 2 –Bayesian additive regression trees

Boosting is a popular machine learning algorithm which combines many so-called weaker learners sequentially and produce a powerful predictive model. Bayesian additive regression trees (BART) [12] is similar to boosting although each tree is constrained by a regularization prior to be a weak learner, and fitting and inference are accomplished via an iterative Bayesian MCMC algorithm that generates samples from a posterior. This nonparametric Bayesian approach allows full posterior inference including point and interval estimates of the unknown regression function. BART requires to specify a prior for the leaf value (*k*) and the number of trees(*m*), which can influence the conservatism of the fitting process. The recommended default values are *k* = 2 and *m* = 50 for achieving good results [12]. We employed the default prior configurations provided in the R package BART, which, as advocated by [12], serve to regularize the fit by constraining individual tree effects to be small. BART has been shown to have very impressive out-of-box performance with minimal tuning [14]. We adopted the same multiple imputations as before for late outcomes. The model evaluation was similar to Bayesian logistic regression although there was a lack of odds ratio due to the nature of a nonparametric model. Similar to Method 1, we utilized ELPD for model comparison.

### Method 3 –frequentist logistic regression

For the early outcomes, logistic regression models were fit, and we computed odds ratios with 95% CI and p-values. We evaluated model performance with AUC, sensitivity, specificity, positive and negative predictive value. The corresponding 95% CIs were computed with 999 nonparametric bootstrap replications. To evaluate standard error and confidence intervals of CV-AUC, a computationally efficient method was utilized [15].

For the late outcomes, 100 multiple imputations were utilized for missing data. For each imputed data set, a logistic regression model was fit, and final models were computed using Rubin’s rule across multiple imputations to assess odds ratios with 95% CI and p-values. Model performances were combined from multiple imputations to generate 95% CI. For each imputed dataset, we computed standard error of CV-AUC following [15] and utilized nonparametric bootstrap-based standard error of AUC, sensitivity, specificity, positive and negative predictive value.

## Results

**Fig 1:**
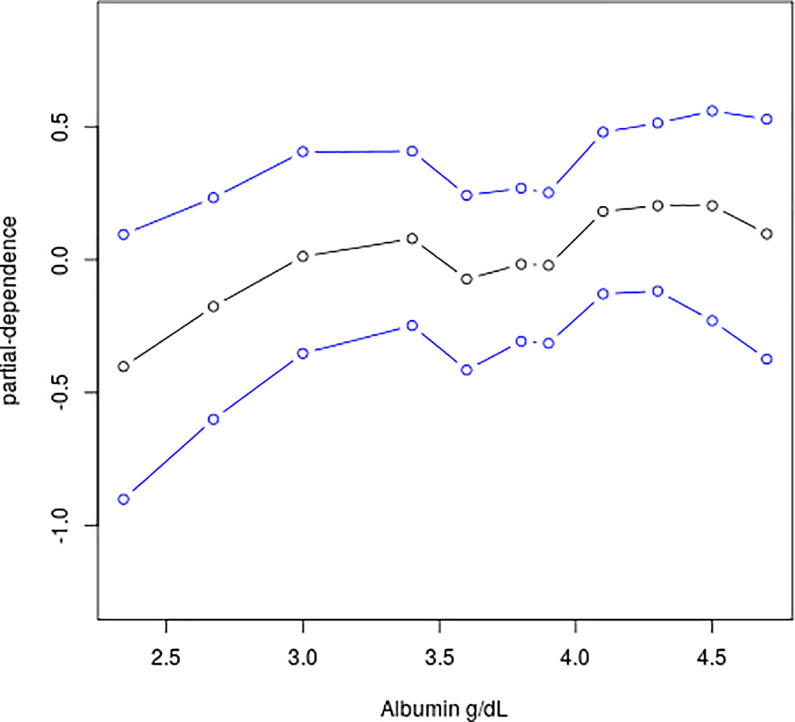
Partial dependent plot for albumin with 95% credible intervals in the BART model of week 4 remission for all patients. The plot summarizes the marginal effect of albumin on the response (probit of the probability estimate) using the posterior median, lower quantile, and upper quantile.

For week 4 CS-free remission with all patients, a baseline total Mayo score less than 10 at diagnosis (OR 1.85, 95% CI 1.11–3.12), proctosigmoiditis (5.37, 1.93–18.97), rectal biopsy eosinophil peak count larger than 32 cells per high-power field (1.74, 1.11–2.74), and relative rectal sparing (4.71, 1.92–13.43) were all associated with increased odds of week 4 CS-free remission from the BLR model (n = 355; Table 1). The BLR model showed fair predictive accuracy (AUC 0.69, 0.67–0.70). For the cohort of the intravenous corticosteroid group using additional medical therapy or colectomy by week 4, the BLR model included baseline total Mayo score larger than or equal to 11 (OR 5.83, 95% CI 1.99–18.91), serum albumin g/dL (0.25, 0.09–0.58), rectal biopsy eosinophil count of less than or equal to 32 cells per high-power field (7.63, 2.50–27.20), and rectal biopsy surface villiform changes (3.29, 1.14–9.96). A strong prediction was achieved, with an AUC of 0.86 (95% CI 0.84–0.87), a cross-validated AUC of 0.83 (0.72–0.92), a specificity of 0.90 (95% CI 0.83–0.97). The BART (S1 Table) and BLR models had similar prediction accuracy while a BART model can detect a nonlinear nonparametric association as evidenced in Fig 1. As albumin level increases, the probability of week 4 remission increases in a nonlinear fashion on a probit scale. There was underestimation of effect estimates in the FLR models in comparison to the BLR models (S5 Table). The FLR models had similar AUCs with wider interval estimates.

**Table 1:** Bayesian multivariable logistic regression models of baseline evaluation associated with week 4 remission and additional therapy/colectomy for patients initially treated with IV steroids.

	Remission, all patients	Remission by Initial Treatment			Additional Therapy/ Colectomy
Odds Ratio (95% CI)	Total (N = 419) #	5-ASA (N = 135)	Oral CS (N = 142)	IV CS (N = 142)	IV CS only (N = 142)
Model sample size (% of total N)	n = 355 (85%)	n = 132 (98%)	n = 123 (87%)	n = 142 (100%)	n = 120 (85%)
Number of events (% of model n)	179 (50%)	73 (55%)	70 (57%)	57 (40%)	32 (27%)
Total Mayo score	Mayo < 10:	-	Mayo < 10:	Mayo < 11:	Mayo ≥ 11:
	1.85 (1.11, 3.12)		3.43 (1.25, 9.81)	2.35 (1.18, 4.80)	5.83 (1.99, 18.91)
Albumin per 1 g/dL	1.38 (0.99, 1.92)	2.11 (1.23, 3.73)	-	-	0.25 (0.09, 0.58)
Proctosigmoiditis	5.37 (1.93, 18.97)	3.15 (1.11, 10.66)	**-**	-	**-**
Rectal biopsy eosinophil peak count /hpf	Count > 32:	-	Count > 32:	-	Count ≤ 32:
	1.74 (1.11, 2.74)		2.86 (1.29, 6.70)		7.63 (2.50, 27.20)
Relative rectal sparing	4.71 (1.92, 13.43)	-	8.55 (1.85, 67.12)	-	-
Rectal biopsy surface villiform changes	-	-	-	-	3.29 (1.14, 9.96)
**Model evaluation**					
AUC	0.69 (0.67, 0.70)	0.66 (0.64, 0.66)	0.70 (0.68, 0.71)	0.60 (0.60, 0.60)	0.86 (0.84, 0.87)
CV-AUC	0.67 (0.59, 0.72)	0.65 (0.59, 0.68)	0.69 (0.58, 0.73)	0.59 (0.54, 0.60)	0.83 (0.72, 0.91)
Sensitivity	0.62 (0.41, 0.76)	0.73 (0.38, 0.95)	0.77 (0.73, 0.90)	0.67 (0.00, 1.00)	0.58 (0.41, 0.69)
Specificity	0.66 (0.48, 0.85)	0.46 (0.20, 0.78)	0.54 (0.26, 0.64)	0.51 (0.00, 1.00)	0.90 (0.83, 0.97)
Positive predictive value	0.66 (0.60, 0.75)	0.63 (0.58, 0.70)	0.70 (0.62, 0.73)	0.46 (0.00, 0.49)	0.69 (0.58, 0.80)
Negative predictive value	0.63 (0.59, 0.67)	0.59 (0.49, 0.74)	0.65 (0.64, 0.67)	0.67 (0.00, 0.71)	0.86 (0.81, 0.89)

# Total N = number evaluable at week 4 and with no protocol violations. AUC = area under the curve. CV-AUC = 10-fold cross validation AUC

Table 2 shows the Bayesian multivariable model of baseline and week 4 risk factors associated with week 12 CS-free remission for all initial therapy groups and additional therapy or colectomy for patients treated with intravenous corticosteroids. For the full cohort (n = 409), predictive factors for corticosteroid-free remission included baseline PUCAI less than 35 and increasing serum albumin by 1 g/dL increments in children younger than 12 years, week 4 clinical remission, which is most unvaryingly associated with corticosteroid-free remission across the full cohort and for each initial treatment group, with the largest effect with intravenous corticosteroids and the smallest effect with mesalamine. Similar conclusions hold for the FLR models (S6 Table). For the cohort of the intravenous corticosteroid group using additional medical therapy or colectomy by week 12, the BLR model included baseline total Mayo score larger than or equal to 11 (OR 2.69, 95% CI 0.98–7.81), rectal biopsy eosinophil count of less than or equal to 32 cells per high-power field (4.79, 1.72–13.98), the existence of surface villiform changes (3.20, 1.14–9.35), and the lack of remission by week 4 (34.47, 8.65–217.75). The predictive accuracy was strong with an AUC of 0.88 (95% CI 0.86–0.89). For week 12 CS-free remission with all patients, there was underestimation of effect estimates in the FLR models in comparison to the BLR models on baseline total Mayo score of 11 or more, haemoglobin of 10 g/dL or more, rectal biopsy eosinophil peak count, rectal biopsy surface villform changes and week 4 remission. AUCs between the BLR, BART (S2 Table) and FLR models were similar with narrower interval estimates for the first two models. The FLR models had slightly larger cross-validated AUCs but wider interval estimates than the BLR models, which in turn had marginal advantages on cross-validated AUCs than the BART models. These models generated similar results of sensitivity, specificity, PPV and NPV.

**Table 2:** Bayesian multivariable logistic regression models of week 12 outcomes by treatment type.

	CS-Free Remission, all patients	CS-Free Remission by Initial Treatment			Additional Therapy/Colectomy
Odds Ratio (95% CI)	Total (N = 409)#	5-ASA (N = 129)	Oral CS (N = 139)	IV CS (N = 141)	IV CS only (N = 141)
p-value					
Model sample size (% of total N)	n = 403 (99%)	n = 116 (90%)	n = 139 (100%)	n = 119 (84%)	n = 119 (84%)
Number of events (% of model n)	140 (35%)	57 (49%)	47 (34%)	26 (22%)	42 (35%)
**Baseline predictors:**					
Lower PUCAI	**PUCAI <35:**	-	**PUCAI < 45:**	*-*	*-*
	2.43 (1.41, 4.27)		4.50 (1.86, 10.96)		
Total Mayo score ≥11	**-**	-	**-**	*-*	2.69 (0.98, 7.81)
Higher albumin per 1g/dL increase (interaction with age)	For **Age < 12**:	**-**	-	**-**	**-**
	3.56 (1.84, 7.48)				
	For **Age** ≥ 12:				
	1.22 (0.79, 1.82)				
Hemoglobin ≥12 g/dL	-	2.22 (0.98, 5.16)	-	**-**	**-**
Rectal biopsy eosinophil peak count ≤32/hpf	-	-	-	**-**	4.79 (1.72, 13.98)
Rectal biopsy surface villiform changes	-	-	-	**No changes:**	**Changes:**
				2.76 (1.04, 8.29)	3.20 (1.14, 9.35)
Week 4 Remission	6.37 (3.90, 10.64)	3.79 (1.71, 8.63)	8.35 (3.43, 23.71)	7.67 (2.87, 23.53)	**No Remission**:
					34.47 (8.65, 217.76)
**Model evaluation**					
AUC	0.78 (0.77, 0.79)	0.70 (0.63, 0.70)	0.78 (0.76, 0.78)	0.77 (0.74, 0.77)	0.88 (0.86, 0.89)
CV-AUC	0.78 (0.73, 0.81)	0.68 (0.55, 0.72)	0.77 (0.74, 0.80)	0.76 (0.58, 0.80)	0.87 (0.80, 0.91)
Sensitivity	0.46 (0.37, 0.65)	0.65 (0.54, 0.74)	0.43 (0.40, 0.85)	0.33 (0.00, 0.62)	0.75 (0.57, 0.86)
Specificity	0.87 (0.74, 0.91)	0.66 (0.47, 0.76)	0.91 (0.58, 0.93)	0.92 (0.86, 1.00)	0.86 (0.74, 0.94)
Positive predictive value	0.66 (0.57, 0.70)	0.65 (0.58, 0.69)	0.74 (0.51, 0.76)	0.29 (0.00, 0.55)	0.76 (0.64, 0.84)
Negative predictive value	0.75 (0.73, 0.80)	0.67 (0.63, 0.69)	0.76 (0.75, 0.88)	0.84 (0.78, 0.89)	0.86 (0.79, 0.91)

#N is the number evaluable at week 12 and with no protocol violations. AUC = area under the curve. CV-AUC = 10-fold cross validation AUC.

For week 52 CS-free remission with all patients, PUCAI score of less than 45, baseline haemoglobin concentration of 10 g/dL or higher plus week 4 remission were associated with week 52 CS-free remission (n = 386; Table 3). The BLR model had moderate diagnostic test power, with an AUC of 0.69 (95% CI 0.68–0.70), a cross-validated AUC of 0.68 (0.66–0.71), and specificity of 82% (95% CI 0.61–0.86). The BART model showed similar prediction results with an AUC of 0.69 (95% CI 0.67–0.70), a cross-validated AUC of 0.68 (0.62–0.72), and specificity of 83% (95% CI 61–88) (S3 Table). There was underestimation of effect estimates in the FLR models in comparison to the BLR models on haemoglobin and week 4 remission (S7 Table). The FLR model had similar prediction accuracy. The BLR and FLR model shared similar effect estimates and interval estimates for biological data on antimicrobial peptide gene signature and the relative abundance of Ruminococcaceae and Sutterella organisms. The addition of biological data to the clinical model improved the diagnostic test power of the BLR model, with an ELPD 6.2 (SE 4.1), an AUC of 0.75 (95% CI 0.72–0.76), a cross-validated AUC of 0.73 (95% CI 0.63–0.81), and specificity of 78% (95% CI 65–91) in the subset with biological data. Similar conclusions hold for the BART model with an ELPD 4.4 (SE 3.6), an AUC of 0.76 (95% CI 0.72–0.79), a cross-validated AUC of 0.69 (95% CI 0.53–0.83), and specificity of 79% (95% CI 65–93). Model accuracy of the BLR and BART models was comparable or better than the FLR models, which obtained the smallest specificity 0.6 (95% CI 0.24–0.96) for patients with biological data in the clinical model. Similarly, the addition of biological data to the clinical model improved the diagnostic test power of the FLR model (p = 0.00038).

**Table 3:** Bayesian multivariable logistic regression models of week 52 corticosteroid-free remission in the per-protocol population.

	All patients in clinical model	Patients with biological data	
	(n = 386; 147 [38%] events)	(n = 177; 69 [39%] events)	
		Clinical model	Clinical and biological model
**Baseline predictors**			
PUCAI score <45	1.80 (1.20, 2.90)	-	-
Haemoglobin ≥10 g/dL (without week 4 remission)	5 (1.8, 17)	7.3 (1.8, 42)	6.34 (1.44, 38.82)
Week 4 remission	11.20 (4.10, 37.00)	16.7 (4.3, 93)	17.25 (4.16, 101.17)
Antimicrobial peptide gene signature	-	-	0.55 (0.38, 0.80)
Ruminococcaceae (560535) OTU log relative abundance	-	-	1.45 (1.04, 2.07)
*Sutterella* (589923) OTU log relative abundance	-	-	0.80 (0.64, 0.99)
**Model evaluation**			
AUC	0.69 (0.68, 0.70)	0.68 (0.68, 0.68)	0.75 (0.72, 0.76)
CV-AUC	0.68 (0.66, 0.71)	0.67 (0.58, 0.68)	0.73 (0.63, 0.81)
Sensitivity	0.38 (0.33, 0.67)	0.48 (0.00, 0.70)	0.51 (0.35, 0.65)
Specificity	0.82 (0.61, 0.86)	0.72 (0.60, 1.00)	0.78 (0.65, 0.91)
Positive predictive value	0.57 (0.52, 0.60)	0.37 (0.00, 0.53)	0.61 (0.53, 0.72)
Negative predictive value	0.69 (0.68, 0.75)	0.71 (0.61, 0.76)	0.72 (0.68, 0.75)
Clinical plus biological model vs clinical model§			
Comparison of ELPD with SE			6.2 (4.1)

AUC = area under the curve. CV-AUC = 10-fold cross validation. §Comparison of the clinical plus biological model with clinical model in the subset of patients with biological data.

For patients with moderate-to-severe disease escalated to anti-TNF*α* therapy by week 52, predictive risk factors included a baseline total Mayo score of 11 or higher. High eosinophil count in rectal biopsy samples, high serum 25(OH)D concentration, haemoglobin concentration of greater than or equal to 10 g/dL, and remission by week 4 were also associated with a lack of escalaton to anti-TNF*α* (n = 232; Table 4). The BLR model had good diagnostic test power, with an AUC of 0.78 (95% CI 0.76–0.80), a cross-validated AUC of 0.76 (0.68–0.84), sensitivity of 60% (95% CI 51–68), and specificity of 84% (95% CI 74–91). The BART model had a close prediction accuracy, with an AUC of 0.78 (95% CI 0.76–0.80), a cross-validated AUC of 0.75 (0.64–0.84), sensitivity of 0.59% (95% CI 0.49–0.68), and specificity of 85% (95% CI 75–93) (S4 Table). The addition of biological data on transport and antimicrobial gene signature and the abundance of an Oscillospira species to the BLR model improved the model prediction with an ELPD of 7.7 (SE 4.8), an AUC of 0.86 (95% CI 0.84–0.88), a cross-validated AUC of 0.84 (0.75–0.92), sensitivity of 69% (95% CI 59–80), and specificity of 84% (95% CI 76–92) in the subset with biological data (n = 118; Table 4). Similarly, the addition of biological data to the BART model improved the model prediction with an ELPD of 9.0 (SE 3.7), an AUC of 0.88 (95% CI 0.84–0.90), a cross-validated AUC of 0.82 (0.68–0.95), sensitivity of 69% (95% CI 54–83), and specificity of 86% (95% CI 76–94) in the subset with biological data (S4 Table). The effect estimates in the FLR and BLR model were similar except for a baseline total Mayo score of 11 or more for which the odds ratio was 4.30 (95% CI 2.18–8.48) compared to 4.48 (95% CI 2.27–9.01) for all patients in the clinical model (n = 232; S8 Table). The FLR models had comparable prediction accuracy compared to the BLR and BART models but with much wider interval estimates of AUC. Finally, the addition of biological data to the FLR model also improved the model prediction (p < 0.00004).

**Table 4:** Bayesian multivariable logistic regression models of escalation to anti-TNFα therapy by week 52 for patients with moderate-to-severe disease.

	All patients in clinical model	Patients with biological data	
	(n = 386; 147 [38%] events)	(n = 177; 69 [39%] events)	
		Clinical model	Clinical and biological model
**Baseline predictors**			
PUCAI score <45	1.80 (1.20, 2.90)	-	-
Haemoglobin ≥10 g/dL (without week 4 remission)	5 (1.8, 17)	7.3 (1.8, 42)	6.34 (1.44, 38.82)
Week 4 remission	11.20 (4.10, 37.00)	16.7 (4.3, 93)	17.25 (4.16, 101.17)
Antimicrobial peptide gene signature	-	-	0.55 (0.38, 0.80)
Ruminococcaceae (560535) OTU log relative abundance	-	-	1.45 (1.04, 2.07)
*Sutterella* (589923) OTU log relative abundance	-	-	0.80 (0.64, 0.99)
**Model evaluation**			
AUC	0.69 (0.68, 0.70)	0.68 (0.68, 0.68)	0.75 (0.72, 0.76)
CV-AUC	0.68 (0.66, 0.71)	0.67 (0.58, 0.68)	0.73 (0.63, 0.81)
Sensitivity	0.38 (0.33, 0.67)	0.48 (0.00, 0.70)	0.51 (0.35, 0.65)
Specificity	0.82 (0.61, 0.86)	0.72 (0.60, 1.00)	0.78 (0.65, 0.91)
Positive predictive value	0.57 (0.52, 0.60)	0.37 (0.00, 0.53)	0.61 (0.53, 0.72)
Negative predictive value	0.69 (0.68, 0.75)	0.71 (0.61, 0.76)	0.72 (0.68, 0.75)
Clinical plus biological model vs clinical model§			
Comparison of ELPD with SE			6.2 (4.1)

AUC = area under the curve. CV-AUC = 10-fold cross validation. §Comparison of the clinical plus biological model with clinical model in the subset of patients with biological data.

## Discussion

In this study, the comparison of Bayesian and frequentist approaches revealed no significant difference in the predictive performance for clinical outcomes in pediatric UC patients. Bayesian advantages include the ability to directly estimate uncertainty, formally incorporate prior probabilities, and be applied with small sample sizes. Conversely, frequentist strengths lie in interpreting probabilities based on long-term event frequency and greater reliability with large samples. While the performance between Bayesian and frequentist models is similar, the interpretation of results differs. For example, credible intervals, unlike confidence intervals, offer more intuitiveness. Bayesian statistics permit direct parameter estimation, allowing for model comparison. While the BLR and BART models had narrower credible intervals of AUC than the FLR models, the corresponding CIs for cross-validated AUC didn’t show the advantage. The CIs for the FLR models were derived from large sample theory to avoid very expensive computations. In a simulation study with sample size *n* = 500, for 95% confidence intervals, the coverage probability was 0.909, below the expected value of 0.95 [15]. In smaller samples as in this study, the coverage probability is expected to be inferior to the nominal level 0.95. In other words, the reported CIs of cross-validated AUC for the FLR models might be narrower than expected.

Predictive modeling can be improved. To predict week 4 CS-free remission for patients with initial treatment intravenous corticosteroids, baseline total Mayo score itself was not strong enough to accurately predict the outcome in the BLR, BART and FLR model, with wide ranges of sensitivity, specificity, PPV and NPV. To predict week 12 CS-free remission with initial treatment intravenous corticosteroids, all three models with baseline total Mayo score, rectal biopsy surface villiform changes and week 4 remission had poor sensitivity and PPV with lower end of interval estimates 0% while substantially higher specificity and NPV. Sensitivity and PPV can be improved by decreasing the prespecified classification cut-off point of 0.5.

It can be interesting to compare the results of the FLR models in S5–S8 Tables with published work, S9 Table and Table 4 in [8], Tables 3 and 4 in [9], respectively. While the effect estimation is similar, the model performance can be quite different with respect to variability and confidence intervals. The variability of a model performance metric such as sensitivity can be estimated based on the asymptotic theory once a model is fixed. The bootstrap procedure in this article, however, perhaps more accurately captures the uncertainty of the modeling since a metric was repeatedly evaluated for logistic regression models with replicated bootstrapped samples. For the late outcomes, multiple imputation could contribute to different results as well. To impute missing data, the default method in SAS was discriminant function for binary variables and linear regression for continuous variables [9]. In R, we chose the default method logistic regression and predictive mean matching, respectively. In addition, multiple imputation is a random process, which could provide different numerical results even with the same imputation method. Utilizing different methods for the same dataset is an important interval validation since data analysis inference such as FLR typically relies on theoretical assumptions, which may fail to hold in practice. With prediction accuracy metrics including sensitivity, specificity, PPV and NPV, we found that even with the same FLR, confidence intervals can vary substantially depending on how to estimate variability.

One limitation of our study is the absence of a variable selection comparison between Bayesian and frequentist methods. Variable selection is crucial in modern data analysis. While our study focused on pre-selected risk factors, providing a straightforward interpretation of effect estimates for BLR and FLR models, a systematic comparison is a potential avenue for future research.

## Conclusion

To conclude, this is the first study comparing BLR, BART and FLR models for a large pediatric cohort with UC. Our study shows that, BLR and BART can be alternative approaches in developing prognostic models in pediatric UC. The BLR models have similar effect estimation compared to the FLR models. The statistically significant associations between the risk factors and early or late outcomes are consistent between all BLR and FLR models. The BLR and BART have similar prediction accuracy and more accurate credible intervals of AUC than the corresponding confidence intervals from the FLR models. Furthermore, the nonlinear nonparametric effect estimate from the BART models can provide more realistic clinical interpretations. The original report (PROTECT study) had already demonstrated predictive factors associated with week 52 corticosteroid-free remission and other outcomes. This manuscript reaffirms that the test characteristics of the different models are like those presented in the original report. However, in terms of clinical utility and interpretation, the models are presented using a Bayesian approach.

## Supporting information

S1 Table BART models of baseline evaluation associated with week 4 remission and additional therapy/colectomy for patients treated with IV steroids. (DOCX)

S2 Table BART models of week 12 outcomes by treatment type. (DOCX)

S3 Table BART models of week 52 corticosteroid-free remission in the per-protocol population. (DOCX)

S4 Table BART models of escalation to anti-TNFα therapy by week 52 for patients with moderate-to-severe disease. (DOCX)

S5 Table Frequentist multivariable logistic regression models of baseline evaluation associated with week 4 remission and additional therapy/colectomy for patients initially treated with IV steroids. (DOCX)

S6 Table Frequentist multivariable logistic regression models of week 12 outcomes by treatment type. (DOCX)

S7 Table Frequentist multivariable logistic regression models of week 52 corticosteroid-free remission in the per-protocol population. (DOCX)

S8 Table Frequentist multivariable logistic regression models of escalation to anti-TNFα therapy by week 52 for patients with moderate-to-severe disease. (DOCX)

## Abbreviations

BLR: Bayesian logistic regression
BART: Bayesian additive regression trees
FLR: Frequentist logistic regression
AUC: Area under the (Receiver Operating Characteristic) Curve
CS: Corticosteroid
UC: Ulcerative colitis
ELPD: Expected log-predictive density
SE: Standard error
