# Supplementary material for: A comparison of Bayesian and frequentist approaches to incorporating clinical and biological information for the prediction of response to standardized pediatric colitis therapy
Source: PLoS One. 2024 Mar 6;19(3):e0295814. doi: 10.1371/journal.pone.0295814 (PMC10917270; doi:10.1371/journal.pone.0295814)
Supplement: S1 Table — (DOCX) [file pone.0295814.s001.docx]

**S1 Table. BART models of baseline evaluation associated with week 4 remission and additional therapy/colectomy for patients treated with IV steroids.**

|  | **CS-Free Remission, all patients** | **CS-Free Remission  by Initial Treatment** | | | **Additional Therapy/ Colectomy** |
| --- | --- | --- | --- | --- | --- |
| **Predictors** | **Total**  **(N=419) #** | **5-ASA**  **(N=135)** | **Oral CS**  **(N=142)** | **IV CS**  **(N=142)** | **IV CS only**  **(N=142)** |
| Model sample size (% of total N) | n=355 (85%) | n=132 (98%) | n=123 (87%) | n=142 (100%) | n=120 (85%) |
| Number of events (% of model n) | 179 (50%) | 73 (55%) | 70 (57%) | 57 (40%) | 32 (27%) |
| Total Mayo score | x | - | x | x | x |
| Albumin per 1 g/dL | x | x | - | - | x |
| Proctosigmoiditis | x | x | **-** | - | **-** |
| Rectal biopsy eosinophil peak count /hpf | x | - | x | - | x |
| Relative rectal sparing | x | - | x | - | - |
| Rectal biopsy surface villiform changes | - | - | - | - | x |
| **Model evaluation** | | | | | |
| AUC | 0.70 (0.67, 0.72) | 0.67 (0.61, 0.71) | 0.70 (0.66, 0.71) | 0.60 (0.60, 0.60) | 0.86 (0.83, 0.89) |
| CV-AUC | 0.66 (0.55, 0.74) | 0.61 (0.39, 0.80) | 0.68 (0.50, 0.74) | 0.59 (0.50, 0.60) | 0.81 (0.65, 0.95) |
| Sensitivity | 0.64 (0.45, 0.78) | 0.74 (0.40, 0.95) | 0.77 (0.73, 0.91) | 0.67 (0.00, 1.00) | 0.56 (0.34, 0.72) |
| Specificity | 0.65 (0.45, 0.82) | 0.47 (0.19, 0.78) | 0.54 (0.19, 0.64) | 0.52 (0.00, 1.00) | 0.91 (0.82, 0.97) |
| Positive predictive value | 0.65 (0.59, 0.73) | 0.64 (0.58, 0.73) | 0.70 (0.60, 0.73) | 0.47 (0.00, 0.49) | 0.69 (0.57, 0.82) |
| Negative predictive value | 0.64 (0.59, 0.68) | 0.61 (0.50, 0.75) | 0.64 (0.62, 0.67) | 0.68 (0.00, 0.71) | 0.85 (0.80, 0.89) |
| # Total N=number evaluable at week 4 and with no protocol violations. x=Predictors used in the models. -=Predictors not used in the models. AUC=area under the curve. CV-AUC=10-fold cross validation. | | | | | |
