# Supplementary material for: A comparison of Bayesian and frequentist approaches to incorporating clinical and biological information for the prediction of response to standardized pediatric colitis therapy
Source: PLoS One. 2024 Mar 6;19(3):e0295814. doi: 10.1371/journal.pone.0295814 (PMC10917270; doi:10.1371/journal.pone.0295814)
Supplement: S2 Table — (DOCX) [file pone.0295814.s002.docx]

**S2 Table. BART models of week 12 outcomes by treatment type.**

|  | **CS-Free Remission, all patients** | **CS-Free Remission by Initial Treatment** | | | **Additional Therapy/Colectomy** |
| --- | --- | --- | --- | --- | --- |
| **Odds Ratio (95% CI)** | **Total (N=409)#** | **5-ASA (N=129)** | **Oral CS (N=139)** | **IV CS (N=141)** | **IV CS only (N=141)** |
| **p-value** |  |  |  |  |  |
| Model sample size (% of total N) | n=403 (99%) | n=116 (90%) | n=139 (100%) | n=119 (84%) | n=119 (84%) |
| Number of events (% of model n) | 140 (35%) | 57 (49%) | 47 (34%) | 26 (22%) | 42 (35%) |
| **Baseline predictors:** |  |  |  |  |  |
| Lower PUCAI | **PUCAI <35:** | - | **PUCAI < 45:** | *-* | *-* |
|  |  |  |  |  |  |
|  | x |  | x |  |  |
| Total Mayo score ≥11 | **-** | - | **-** | *-* | x |
|  |  |  |  |  |  |
| Higher albumin per 1g/dL increase |  | **-** | - | **-** | **-** |
|  |  |  |  |  |  |
|  |  |  |  |  |  |
|  | x |  |  |  |  |
|  |  |  |  |  |  |
|  |  |  |  |  |  |
| Age ≥ 12 | x |  |  |  |  |
| Hemoglobin ≥12 g/dL | - | x | - | **-** | **-** |
|  |  |  |  |  |  |
| Rectal biopsy eosinophil peak count ≤32/hpf | - | - | - | **-** | x |
|  |  |  |  |  |  |
| Rectal biopsy surface villiform changes | - | - | - | **No changes:** | **Changes:** |
|  |  |  |  | x | x |
|  |  |  |  |  |  |
| Week 4 Remission | x | x | x | x | **No Remission**: |
|  |  |  |  |  | x |
| **Model evaluation** | | | | | |
| AUC | 0.78 (0.77, 0.79) | 0.69 (0.66, 0.70) | 0.78 (0.76, 0.78) | 0.77 (0.70, 0.77) | 0.88 (0.85, 0.90) |
| CV-AUC | 0.76 (0.69, 0.81) | 0.67 (0.54, 0.75) | 0.77 (0.71, 0.81) | 0.74 (0.54, 0.82) | 0.86 (0.76, 0.92) |
| Sensitivity | 0.50 (0.35, 0.71) | 0.66 (0.54, 0.91) | 0.43 (0.40, 0.85) | 0.37 (0.00, 0.62) | 0.73 (0.50, 0.86) |
| Specificity | 0.84 (0.70, 0.91) | 0.65 (0.32, 0.76) | 0.91 (0.58, 0.93) | 0.91 (0.86, 1.00) | 0.87 (0.75, 0.95) |
| Positive predictive value | 0.63 (0.55, 0.69) | 0.65 (0.57, 0.69) | 0.74 (0.51, 0.76) | 0.34 (0.00, 0.55) | 0.76 (0.65, 0.84) |
| Negative predictive value | 0.76 (0.72, 0.83) | 0.67 (0.63, 0.79) | 0.76 (0.75, 0.88) | 0.85 (0.78, 0.89) | 0.86 (0.78, 0.91) |
| #N is the number evaluable at week 12 and with no protocol violations. x=Predictors used in the models. - =Predictors not used in the models. AUC=area under the curve. CV-AUC=10-fold cross validation AUC | | | | | |
