# Supplementary material for: A comparison of Bayesian and frequentist approaches to incorporating clinical and biological information for the prediction of response to standardized pediatric colitis therapy
Source: PLoS One. 2024 Mar 6;19(3):e0295814. doi: 10.1371/journal.pone.0295814 (PMC10917270; doi:10.1371/journal.pone.0295814)
Supplement: S3 Table — (DOCX) [file pone.0295814.s003.docx]

**S3 Table. BART models of week 52 corticosteroid-free remission in the per-protocol population.**

|  | **All patients in clinical model** | **Patients with biological data** | |
| --- | --- | --- | --- |
|  | **(n=364#; 137 [38%] events)** | **(n=170; 68 [40%] events)** | |
|  |  | **Clinical model** | **Clinical and biological model** |
| **Baseline predictors** |  |  |  |
| PUCAI score <45 | x | - | - |
| Haemoglobin ≥10 g/dL (without week 4 remission) | x | x | x |
| Week 4 remission | x | x | x |
| Antimicrobial peptide gene signature | - | - | x |
| Ruminococcaceae (560535) OTU log relative abundance | - | - | x |
| *Sutterella* (589923) OTU log relative abundance | - | - | x |
| **Model evaluation** | | | |
| AUC | 0.69 (0.67, 0.70) | 0.68 (0.68, 0.68) | 0.76 (0.72, 0.79) |
| CV-AUC | 0.68 (0.62, 0.72) | 0.67 (0.59, 0.68) | 0.69 (0.53, 0.83) |
| Sensitivity | 0.38 (0.12, 0.67) | 0.46 (0.00, 0.70) | 0.53 (0.30, 0.71) |
| Specificity | 0.83 (0.61, 0.88) | 0.73 (0.60, 1.00) | 0.79 (0.65, 0.93) |
| Positive predictive value | 0.57 (0.39, 0.60) | 0.36 (0.00, 0.53) | 0.62 (0.54, 0.74) |
| Negative predictive value | 0.69 (0.62, 0.75) | 0.71 (0.61, 0.76) | 0.73 (0.67, 0.79) |
| Clinical plus biological model vs clinical model§ | | | |
| Comparison of ELPD with SE |  |  | 4.4 (3.6) |
| #The per-protocol population excludes participants who discontinued the study without additional therapy or colectomy or who had protocol violations. x=Predictors used in the models. - =Predictors not used in the models. AUC=area under the curve. CV-AUC=10-fold cross validation AUC. §Comparison of the clinical plus biological model with clinical model in the subset of patients with biological data. | | | |
