# Supplementary material for: A comparison of Bayesian and frequentist approaches to incorporating clinical and biological information for the prediction of response to standardized pediatric colitis therapy
Source: PLoS One. 2024 Mar 6;19(3):e0295814. doi: 10.1371/journal.pone.0295814 (PMC10917270; doi:10.1371/journal.pone.0295814)
Supplement: S4 Table — (DOCX) [file pone.0295814.s004.docx]

**S4 Table. BART models of escalation to anti-TNFα therapy by week 52 for patients with moderate-to-severe disease.**

|  | **All patients in clinical model** | **Patients with biological data** | |
| --- | --- | --- | --- |
|  | **(n=232#; 94 [41%] events)** | **(n=118; 46 [39%] events)** | |
|  |  | **Clinical model** | **Clinical plus biological model** |
| Total Mayo score ≥11 | x | - | - |
| Rectal biopsy eosinophil peak count >32 per hpf | x | - | - |
| Higher 25-hydroxyvitamin D concentration, per increase in category | x | x | x |
| Haemoglobin ≥10 g/dL | x | x | x |
| Week 4 remission | x | x | x |
| Transport and antimicrobial gene signature | - | - | x |
| *Oscillospira* (581079) OTU log relative abundance | - | - | x |
| **Model evaluation** | | | |
| AUC | 0.78 (0.76, 0.80) | 0.79 (0.76, 0.81) | 0.88 (0.84, 0.90) |
| CV-AUC | 0.75 (0.64, 0.84) | 0.76 (0.61, 0.86) | 0.82 (0.68, 0.95) |
| Sensitivity | 0.59 (0.49, 0.68) | 0.55 (0.35, 0.80) | 0.69 (0.54, 0.83) |
| Specificity | 0.85 (0.75, 0.93) | 0.83 (0.62, 0.97) | 0.86 (0.76, 0.94) |
| Positive predictive value | 0.73 (0.64, 0.83) | 0.70 (0.57, 0.89) | 0.76 (0.67, 0.86) |
| Negative predictive value | 0.75 (0.72, 0.78) | 0.75 (0.69, 0.83) | 0.81 (0.76, 0.88) |
| Clinical plus biological model vs clinical model¶ | | | |
| Comparison of ELPD with SE |  |  | 9.0 (3.7) |
| #Of 237 patients with moderate-to-severe disease who were evaluable at week 52, the per-protocol population (n=232) excludes five participants who had protocol violations. x=Predictors used in the models; - =Predictors not used in the models. AUC=area under the curve. CV-AUC=10-fold cross validation AUC. ¶Comparison of the clinical plus biological model with clinical model in the subset of patients with biological data. | | | |
