# Supplementary material for: A comparison of Bayesian and frequentist approaches to incorporating clinical and biological information for the prediction of response to standardized pediatric colitis therapy
Source: PLoS One. 2024 Mar 6;19(3):e0295814. doi: 10.1371/journal.pone.0295814 (PMC10917270; doi:10.1371/journal.pone.0295814)
Supplement: S5 Table — (DOCX) [file pone.0295814.s005.docx]

**S5 Table. Frequentist multivariable logistic regression models of baseline evaluation associated with week 4 remission and additional therapy/colectomy for patients initially treated with IV steroids.**

|  | **CS-Free Remission, all patients** | **CS-Free Remission  by Initial Treatment** | | | **Additional Therapy/ Colectomy** |
| --- | --- | --- | --- | --- | --- |
| **Odds Ratio (95% CI)**  **p-value** | **Total**  **(N=419) #** | **5-ASA**  **(N=135)** | **Oral CS**  **(N=142)** | **IV CS**  **(N=142)** | **IV CS only**  **(N=142)** |
| Model sample size (% of total N) | n=355 (85%) | n=132 (98%) | n=123 (87%) | n=142 (100%) | n=120 (85%) |
| Number of events (% of model n) | 179 (50%) | 73 (55%) | 70 (57%) | 57 (40%) | 32 (27%) |
| Total Mayo score | Mayo < 10:  1.83 (1.10, 3.06)  0.020 | - | Mayo < 10:  3.29  (1.22, 8.85)  0.019 | Mayo < 11:  2.33  (1.15, 4.69)  0.018 | Mayo ≥ 11:  5.50  (1.85, 16.31)  0.002 |
| Albumin per 1 g/dL | 1.37  (0.99, 1.90)  0.058 | 2.06  (1.20, 3.54)  0.009 | - | - | 0.26  (0.11, 0.63)  0.003 |
| Proctosigmoiditis | 5.00  (1.64, 15.23)  0.005 | 3.02  (1.01, 9.01)  0.048 | **-** | - | **-** |
| Rectal biopsy eosinophil peak count /hpf | Count > 32:  1.73  (1.10, 2.71)  0.018 | - | Count > 32:  2.82  (1.24, 6.41)  0.013 | - | Count ≤ 32:  7.04  (2.21, 22.41)  0.001 |
| Relative rectal sparing | 4.49  (1.75, 11.47)  0.002 | - | 7.42  (1.41, 39.11)  0.018 | - | - |
| Rectal biopsy surface villiform changes | - | - | - | - | 3.16  (1.09, 9.14)  0.034 |
| **Model evaluation** | | | | | |
| AUC | 0.70 (0.65, 0.76) | 0.66 (0.57, 0.76) | 0.71 (0.63, 0.80) | 0.60 (0.52, 0.68) | 0.87 (0.80, 0.95) |
| CV-AUC | 0.68 (0.62, 0.74) | 0.65 (0.56, 0.75) | 0.69 (0.58, 0.81) | 0.60 (0.47, 0.72) | 0.84 (0.76, 0.92) |
| Sensitivity | 0.60 (0.42, 0.77) | 0.77 (0.35, 0.95) | 0.74 (0.65, 0.93) | 0.68 (0.00, 1.00) | 0.59 (0.37, 0.79) |
| Specificity | 0.71 (0.49, 0.85) | 0.42 (0.12, 0.82) | 0.62 (0.23, 0.76) | 0.52 (0.00, 1.00) | 0.92 (0.84, 0.97) |
| Positive predictive value | 0.68 (0.61, 0.74) | 0.62 (0.55, 0.73) | 0.72 (0.61, 0.82) | 0.49 (0.00, 0.60) | 0.73 (0.58, 0.87) |
| Negative predictive value | 0.64 (0.58, 0.70) | 0.60 (0.44, 0.71) | 0.65 (0.54, 0.79) | 0.71 (0.00, 0.83) | 0.86 (0.80, 0.93) |
| # Total N=number evaluable at week 4 and with no protocol violations. AUC=area under the curve. CV-AUC=10-fold cross validation AUC. | | | | | |
