# Supplementary material for: A comparison of Bayesian and frequentist approaches to incorporating clinical and biological information for the prediction of response to standardized pediatric colitis therapy
Source: PLoS One. 2024 Mar 6;19(3):e0295814. doi: 10.1371/journal.pone.0295814 (PMC10917270; doi:10.1371/journal.pone.0295814)
Supplement: S6 Table — (DOCX) [file pone.0295814.s006.docx]

**S6 Table. Frequentist multivariable logistic regression models of week 12 outcomes by treatment type.**

|  | **CS-Free Remission, all patients** | **CS-Free Remission  by Initial Treatment** | | | **Additional Therapy/ Colectomy** |
| --- | --- | --- | --- | --- | --- |
| **Odds Ratio (95% CI)** | **Total (N=409)#** | **5-ASA (N=129)** | **Oral CS (N=139)** | **IV CS (N=141)** | **IV CS only (N=141)** |
| **p-value** |  |  |  |  |  |
| Model sample size (% of total N) | n=403 (99%) | n=116 (90%) | n=139 (100%) | n=119 (84%) | n=119 (84%) |
| Number of events (% of model n) | 140 (35%) | 57 (49%) | 47 (34%) | 26 (22%) | 42 (35%) |
| **Baseline predictors:** |  |  |  |  |  |
| Lower PUCAI | **PUCAI <35:** | - | **PUCAI < 45:** | *-* | *-* |
|  | 2.44 (1.41, 4.22) |  | 4.38 (1.81, 10.60) |  |  |
|  | 0.001 |  | 0.001 |  |  |
| Total Mayo score ≥11 | **-** | - | **-** | *-* | 2.59 (0.93, 7.21) |
|  |  |  |  |  | 0.068 |
| Higher albumin per 1g/dL increase (interaction with age) | For **Age < 12**: | **-** | - | **-** | **-** |
|  | 4.05 (1.90, 8.64) |  |  |  |  |
|  | 0.0003 |  |  |  |  |
|  | For **Age** ≥ 12: |  |  |  |  |
|  | 1.13 (0.74, 1.71) |  |  |  |  |
|  | 0.57 |  |  |  |  |
| Hemoglobin ≥12 g/dL | - | 2.19 (0.96, 4.97) | - | **-** | **-** |
|  |  | 0.062 |  |  |  |
| Rectal biopsy eosinophil peak count ≤32/hpf | - | - | - | **-** | 4.55 (1.62, 12.78) |
|  |  |  |  |  | 0.004 |
| Rectal biopsy surface villiform changes | - | - | - | **No changes:** | **Changes:** |
|  |  |  |  | 2.71 (0.97, 7.56) | 3.05 (1.09, 8.56) |
|  |  |  |  | 0.057 | 0.034 |
| Week 4 Remission | 6.26 (3.79, 10.35) | 3.69 (1.67, 8.15) | 8.02 (3.11, 20.70) | 7.48 (2.67, 20.96) | **No Remission**: |
|  | < 0.0001 | 0.001 | <0.0001 | 0.0001 | 30.28 (6.36, 144.21) |
|  |  |  |  |  | < 0.0001 |
| **Model evaluation** | | | | | |
| AUC | 0.79 (0.74, 0.83) | 0.70 (0.61, 0.79) | 0.78 (0.70, 0.86) | 0.77 (0.67, 0.87) | 0.89 (0.82, 0.95) |
| CV-AUC | 0.78 (0.73, 0.83) | 0.70 (0.58, 0.82) | 0.79 (0.68, 0.89) | 0.78 (0.68, 0.89) | 0.88 (0.81, 0.95) |
| Sensitivity | 0.44 (0.34, 0.71) | 0.72 (0.45, 0.86) | 0.40 (0.26, 0.88) | 0.62 (0.00, 0.80) | 0.71 (0.60, 0.89) |
| Specificity | 0.89 (0.74, 0.93) | 0.61 (0.44, 0.85) | 0.94 (0.63, 0.98) | 0.86 (0.81, 1.00) | 0.91 (0.77, 0.96) |
| Positive predictive value | 0.68 (0.58, 0.76) | 0.64 (0.56, 0.79) | 0.76 (0.56, 0.92) | 0.55 (0.00, 0.73) | 0.81 (0.67, 0.90) |
| Negative predictive value | 0.75 (0.71, 0.82) | 0.69 (0.58, 0.80) | 0.75 (0.68, 0.89) | 0.89 (0.76, 0.94) | 0.85 (0.81, 0.94) |
| #N is the number evaluable at week 12 and with no protocol violations. AUC=area under the curve.  CV-AUC=10-fold cross validation AUC. | | | | | |
