# Supplementary material for: A comparison of Bayesian and frequentist approaches to incorporating clinical and biological information for the prediction of response to standardized pediatric colitis therapy
Source: PLoS One. 2024 Mar 6;19(3):e0295814. doi: 10.1371/journal.pone.0295814 (PMC10917270; doi:10.1371/journal.pone.0295814)
Supplement: S7 Table — (DOCX) [file pone.0295814.s007.docx]

**S7 Table. Frequentist multivariable logistic regression models of week 52 corticosteroid-free remission in the per-protocol population.**

|  | **All patients in clinical model** | | **Patients with biological data** | | | |
| --- | --- | --- | --- | --- | --- | --- |
|  | **(n=386; 147 [38%] events)** | | **(n=177; 69 [39%] events)** | | | |
|  | **Estimate** | **p value** | **Clinical model** | | **Clinical and biological model** | |
|  |  |  | **Estimate** | **p value** | **Estimate** | **p value** |
| **Baseline predictors** |  |  |  |  |  |  |
| PUCAI score <45 | 1.82 (1.16, 2.86) | 0.009 | - | - | - | - |
| Haemoglobin ≥10 g/dL (without week 4 remission) | 4.77 (1.56, 14.57) | 0.006 | 6.74 (1.43, 31.82) | 0.016 | 5.71 (1.15, 28.43) | 0.034 |
| Week 4 remission | 10.59 (3.60, 31.17) | <0.001 | 15.05 (3.34, 67.84) | <0.001 | 14.92 (3.15, 70.75) | 0.001 |
| Antimicrobial peptide gene signature | - | - | - | - | 0.57 (0.39, 0.82) | 0.003 |
| Ruminococcaceae (560535) OTU log relative abundance | - | - | - | - | 1.43 (1.02, 2.01) | 0.037 |
| *Sutterella* (589923) OTU log relative abundance | - | - | - | - | 0.80 (0.65, 1.00) | 0.049 |
| **Model evaluation** | | | | | | |
| AUC | 0.69 (0.64, 0.74) | - | 0.68 (0.60, 0.74) | - | 0.75 (0.68, 0.82) | - |
| CV-AUC | 0.68 (0.61, 0.74) |  | 0.68 (0.57, 0.77) |  | 0.74 (0.66, 0.81) |  |
| Sensitivity | 0.33 (0.04, 0.63) |  | 0.70 (0.05, 1.00) |  | 0.51 (0.32, 0.69) |  |
| Specificity | 0.86 (0.69, 1.00) |  | 0.60 (0.24, 0.96) |  | 0.79 (0.67, 0.92) |  |
| Positive predictive value | 0.60 (0.40, 0.80) |  | 0.53 (0.02, 1.00) |  | 0.61 (0.50, 0.71) |  |
| Negative predictive value | 0.68 (0.60, 0.75) |  | 0.76 (0.62, 0.89) |  | 0.72 (0.64, 0.79) |  |
| Clinical plus biological model vs clinical model§ | | | | | | |
| Likelihood ratio test |  |  |  |  |  | 0.00038 |
| AUC=area under the curve. CV-AUC=10-fold cross validation AUC. §Comparison of the clinical plus biological model with clinical model in the subset of patients with biological data. | | | | | | |
