# Supplementary material for: A comparison of Bayesian and frequentist approaches to incorporating clinical and biological information for the prediction of response to standardized pediatric colitis therapy
Source: PLoS One. 2024 Mar 6;19(3):e0295814. doi: 10.1371/journal.pone.0295814 (PMC10917270; doi:10.1371/journal.pone.0295814)
Supplement: S8 Table — (DOCX) [file pone.0295814.s008.docx]

**S8 Table. Frequentist multivariable logistic regression models of escalation to anti-TNFα therapy by week 52 for patients with moderate-to-severe disease.**

|  | **All patients in clinical model** | | **Patients with biological data** | | | |
| --- | --- | --- | --- | --- | --- | --- |
|  | **(n=232; 94 [41%] events)** | | **(n=118; 46 [39%] events)** | | | |
|  | **Estimate** | **p value** | **Clinical model** | | **Clinical plus biological model** | |
|  |  |  | **Estimate** | **p value** | **Estimate** | **p value** |
| **Baseline predictors** | | | | | | |
| Total Mayo score ≥11 | 4.30 (2.18, 8.48) | <0.001 | - | - | - | - |
| Rectal biopsy eosinophil peak count >32 per hpf | 0.40 (0.20, 0.78) | 0.008 | - | - | - | - |
| Higher 25-hydroxyvitamin D concentration, per increase in category | 0.55 (0.33, 0.90) | 0.018 | 0.40 (0.20, 0.80) | 0.009 | 0.32 (0.14, 0.73) | 0.007 |
| Haemoglobin ≥10 g/dL | 0.49 (0.25, 0.93) | 0.031 | 0.29 (0.11, 0.76) | 0.013 | 0.33 (0.11, 0.98) | 0.045 |
| Week 4 remission | 0.37 (0.19, 0.69) | 0.002 | 0.16 (0.06, 0.41) | <0.001 | 0.20 (0.07, 0.59) | 0.003 |
| Transport and antimicrobial gene signature | - | - | - | - | 0.30 (0.15, 0.60) | 0.001 |
| *Oscillospira* (581079) OTU log relative abundance | - | - | - | - | 0.64 (0.44, 0.93) | 0.019 |
| **Model evaluation** | | | | | | |
| AUC | 0.79 (0.72, 0.85) | - | 0.80 (0.71, 0.87) | - | 0.87 (0.79, 0.93) | - |
| CV-AUC | 0.77 (0.70, 0.83) |  | 0.80 (0.68, 0.88) |  | 0.86 (0.77, 0.92) |  |
| Sensitivity | 0.60 (0.47, 0.72) |  | 0.62 (0.40, 0.84) |  | 0.69 (0.53, 0.84) |  |
| Specificity | 0.86 (0.76, 0.96) |  | 0.82 (0.69, 0.95) |  | 0.86 (0.77, 0.94) |  |
| Positive predictive value | 0.74 (0.63, 0.86) |  | 0.69 (0.55, 0.82) |  | 0.75 (0.64, 0.87) |  |
| Negative predictive value | 0.76 (0.69, 0.82) |  | 0.77 (0.68, 0.86) |  | 0.81 (0.73, 0.89) |  |
| Clinical plus biological model vs clinical model¶ | | | | | | |
| Likelihood ratio test |  |  |  |  |  | < 0.00004 |
| AUC=area under the curve. CV-AUC=10-fold cross validation AUC. ¶Comparison of the clinical plus biological model with clinical model in the subset of patients with biological data. | | | | | | |
